# Compositional Features of the “Kweli” Red Raspberry and Its Antioxidant and Antimicrobial Activities

**DOI:** 10.3390/foods9111522

**Published:** 2020-10-23

**Authors:** Ana Luísa Vara, José Pinela, Maria Inês Dias, Jovana Petrović, António Nogueira, Marina Soković, Isabel C. F. R. Ferreira, Lillian Barros

**Affiliations:** 1 Centro de Investigação de Montanha (CIMO), Instituto Politécnico de Bragança, Campus de Santa Apolónia, 5300-253 Bragança, Portugal; anavara2009@hotmail.com (A.L.V.); maria.ines@ipb.pt (M.I.D.); ajmnogueira@ipb.pt (A.N.); iferreira@ipb.pt (I.C.F.R.F.); 2 Institute for Biological Research “Siniša Stanković”-National Institute of Republic of Serbia, University of Belgrade, Bulevar Despota Stefana 142, 11000 Belgrade, Serbia; jovana0303@ibiss.bg.ac.rs (J.P.); mris@ibiss.bg.ac.rs (M.S.)

**Keywords:** *Rubus idaeus* L., nutritional composition, vitamins, anthocyanins, antioxidant activity, antimicrobial activity

## Abstract

Red raspberries (*Rubus idaeus* L.) are increasingly popular foods in contemporary diets due to their freshness, nutritional value and health claims. Among the existing cultivars, “Kweli” is one of the most productive and widely cultivated. In this study, the nutritional value and chemical composition of “Kweli” red raspberry were characterized by the official method of food analysis and chromatographic techniques, and its antioxidant and antimicrobial activities were tested against biological/biochemical oxidizable substrates and foodborne bacteria and fungi strains, respectively. Carbohydrates (including fructose and glucose, 14.3 and 12.6 g/100 g dw, respectively), proteins (6.8 g/100 g dw), and ashes (3.90 g/100 g dw) were major constituents. The fat content was quite low and constituted mainly by unsaturated fatty acids (58.3%), with a predominance of oleic acid. Fresh red raspberry also contained high levels of citric (2.7 g/100 g) and ascorbic (17 mg/100 g) acids. The anthocyanins (4.51 mg/g extract) cyanidin-*O*-hexoside and mostly cyanidin-*O*-sophoroside were identified in the red raspberry hydroethanolic extract, which was able to inhibit thiobarbituric acid reactive substances (TBARS) formation (EC_50_ of 122 µg/mL), oxidative hemolysis (IC_50_ of 298 µg/mL), and β-carotene bleaching (EC_50_ of 18.7 µg/mL). In turn, the extract was more effective than the food additive E224 against *Bacillus cereus*. All these results highlighted the nutritional quality of “Kweli” red raspberry and showed some compositional differences in relation to other cultivars. Therefore, its inclusion in a daily diet can be helpful to obtain nutrients and antioxidants and bring health benefits.

## 1. Introduction

Raspberries are edible fruits of different *Rubus* species of the Rosaceae family increasingly popular in daily diets. These fruits present different colors, including red and yellow (*Rubus idaeus* L.), purple (*R. neglectus* Peck.), and black (*R. occidentalis* L.), and are usually packaged and sold directly to the consumer; dehydrated for granola and trail mixes, as well as for long-distance transport; or processed into jam, jelly, dessert topping, ice-cream, and yogurt, among other foods, and thus it is possible to find many raspberry-containing products on supermarket shelves [1,2]. In 2018, the production of raspberries worldwide reached 870,209 tons in an area of 124,971 ha, and Russia was the leading producer, with 19% of the world total [3]. The demand for raspberries has risen sharply in Europe and North America [4], mainly due to its freshness, organoleptic features, nutritional value, and health claims.

Red raspberries belong to the subgenus *Ideobatus*, which comprises around 200 species, and are the hardiest and the most commonly grown type. Unlike blackberries (*Rubus* spp., subgenus *Rubus*), which are solid, raspberries are hollow when removed from the plant, where a finger-shaped receptacle remains [2]. Over the past few years, breeding programs have launched new red raspberry cultivars on the market, with enhanced resistance to diseases and desirable quality traits [5,6,7]. These can be divided into two types—floricane-fruiting cultivars such as “Tulameen”, “Willamette”, and “Meeker”, whose first-year primocanes are only vegetative and second-year floricanes bear a crop in early summer, and primocane-fruiting cultivars such as “Autumn Bliss”, “Heritage”, and “Kweli”, which produce a large amount of fruits at the top of the primocanes in the fall and can be double cropped [6,8]. Among primocanes, the “Kweli” is an economically important and high-yielding early fruiting cultivar created by the Advanced Berry Breeding in the Netherlands, and is one of the most widely cultivated worldwide [6,7,9]. This cultivar is vigorous and develops sides at all levels; that is why it is important to reduce the number of growing primocanes to keep the crop open and compact and facilitate harvesting operations [6,7,9]. The fruit is round, large (usually weigh 5 g or more), and dry; has a good flavor; and has a long shelf-life of 10 days or more, which enables transport over longer distances and, consequently, enables it to reach new markets. This cultivar tolerates high temperatures and is suitable for growing in moderate and Mediterranean climates [7].

From a nutritional point of view, red raspberries have been described as containing vitamins, minerals, soluble fibre, sugars, citric acid, and phenolic compounds [10,11,12,13,14,15,16]. Anthocyanins are of particular interest in these fruits, since these pigments provide the characteristic red-purple color, as well as bioactive properties [5,6,17,18]. Depending on the cultivar, cyanidin 3–*O*–glucoside, and cyanidin 3–*O*–sophoroside have been reported as the major anthocyanins [18,19,20,21]. All these constituents not only determine the sensory characteristics of red raspberry, but also its health-promoting effects. Whole raspberry and extracts have been studied for their therapeutic potential for improving disease biomarkers and pathological conditions [17,22,23], which claim that the dietary intake of raspberries has a positive impact on human health.

In the particular case of “Kweli”, the study of Andrianjaka-Camps et al. [6] described high levels of ellagitannins, anthocyanins, and vitamin C in this cultivar, and also in “Autumn Bliss” and “Himbo Top” grown in Conthey, Switzerland. In addition, the authors related the phytochemical content to the greater antioxidant activity measured through the ferric reducing antioxidant power (FRAP) and the oxygen radical absorption capacity (ORAC). Anjos et al. [21] studied the “Kweli” and “Tulameen” cultivars and detected a greater quantity of ellagitannins [galloyl-bis-HHDP-glucose]*^n^* and ellagic acid derivatives in “Kweli”, as well as a higher total soluble solids content, while the total acidity did not change significantly when grown under conventional agricultural practices in Braga, Portugal. In addition, the anthocyanin (cyanidin 3–*O*–sophoroside and cyanidin 3–*O*–glucoside) levels in “Kweli” were not affected by the adopted agronomic practices and, with organic farming, the polyphenol content increased for “Kweli” but decreased for “Tulameen”. To the best of the authors’ knowledge, there are no other studies describing the nutritional composition of the “Kweli” cultivar.

Therefore, due to the lack of information about “Kweli” and knowing that the raspberries’ composition and bioactivity are affected by a number of factors, such as the cultivar and edaphoclimatic conditions of the growing sites, among others [6,10,12,14,21], it becomes relevant to assess the nutritional quality of this widely cultivated and economically important cultivar. Hence, this study was performed to characterize the detailed nutritional (proximate composition, free sugars, organic acids, tocopherols, and fatty acids) and anthocyanin composition of the “Kweli” red raspberry gown in northern Portugal and assess its in vitro antioxidant and antimicrobial activities against biological/biochemical oxidizable substrates and foodborne bacteria and fungi strains, respectively.

## 2. Materials and Methods

### 2.1. Chemicals and Standards

All the standard compounds used for chromatographic quantifications (melezitose, fructose, glucose, sucrose, trehalose, raffinose, ascorbic, citric, and fumaric acids, 47885-U, α-, β-, γ-, and δ-tocopherols, from Sigma-Aldrich, St. Louis, MO, USA; tocol, from Matreya, Pleasant Gap, PA, USA; chlorogenic and caffeic acids and cyanidin-3-*O*-glucoside, from Extrasynthèse, Genay, France) and bioactivity assays (6-hydroxy-2,5,7,8-tetramethylchroman-2-carboxylic acid (trolox), from Sigma-Aldrich) had a purity level of at least 95%. High-performance liquid chromatography (HPLC)-grade formic acid, acetonitrile, and trifluoroacetic acid (TFA) were purchased from Fisher Scientific, Lisbon, Portugal. 2,2′-Azobis(2-methylpropionamidine) dihydrochloride (AAPH) was acquired from Sigma-Aldrich. All the other reagents were of analytical grade and purchased from common sources.

### 2.2. Plant Material

Red raspberries of the cultivar “Kweli” were cultivated by the company “Ponto Agrícola Unipessoal, Lda” in Tabuado, Marco de Canaveses, Portugal, where the certified red raspberry genotype is produced in 200 m tunnel systems with trellis for support. Fresh fruits at commercial maturity, hand-harvested in September 2018, were transported to the laboratory to be immediately frozen and lyophilized (FreeZone 4.5, Labconco, Kansas City, MO, USA), reduced to a fine powder (~20 mesh), and homogenized to obtain a representative sample that was kept at −20 °C until analysis.

### 2.3. Compositional Analysis

#### 2.3.1. Proximate Composition and Energy

The red raspberry sample was analyzed for moisture, protein, fat, and ash contents following the procedures of the Association of Official Analytical Chemists (AOAC) [24]. Briefly, the crude protein content (N × 6.25) was estimated by the macro-Kjeldahl method, using an automatic distillation and titration unit (Pro-Nitro-A, JP Selecta, Barcelona); the crude fat content was determined by Soxhlet extraction with petroleum ether; and the ash content was determined by incineration in a muffle furnace at 550 ± 15 °C. Total carbohydrate content was estimated by difference. The results were given as g per 100 g of the fresh and dry weight (fw and dw, respectively).

The energy value was calculated as follows: 4 × (g protein + g carbohydrates) + 9 × (g fat) [25] and given as kcal per 100 g of fw and dw.

#### 2.3.2. Free Sugars

Free sugars were analyzed in a HPLC system (Knauer, Smartline system 1000, Berlin, Germany) coupled with a refractive index detector (Smartline System 1000), using the internal standard method previously described by Pinela et al. [26]. Briefly, the sample (~1 g) was spiked with melezitose (internal standard, 5 mg/mL) and extracted with 40 mL of 80% ethanol at 80 °C for 30 min. The mixture was centrifuged at 15,000× *g* (Centurion K24OR-2003 refrigerated centrifuge) for 10 min and the supernatant was concentrated under reduced pressure (rotary evaporator Büchi R-210, Flawil, Switzerland) and defatted with ethyl ether. The residue was then dissolved in 5 mL of water and filtered through 0.2 µm nylon filters. Chromatographic separation was achieved on a Eurospher 100-5 NH_2_ column (4.6 × 250 mm, 5 µm, Knauer) operating at 35 °C (7971R Grace oven). The mobile phase was acetonitrile/deionized water (70:30, *v/v*). Data were recorded and processed using the Clarity 2.4 software (DataApex, Prague, Czech Republic) and the results were expressed as g per 100 g of fw and dw.

#### 2.3.3. Organic Acids

Organic acids were analyzed by ultra-fast liquid chromatography (Shimadzu 20A series, Shimadzu Corporation, Kyoto, Japan) coupled to a photodiode array detector (UPLC-PDA) operating in the optimized conditions described by Pereira et al. [27]. Briefly, the sample (~1 g) was stirred with 25 mL of meta-phosphoric acid for 45 min and filtered, first through Whatman No. 4 paper and then through 0.2 µm nylon filters. Chromatographic separation was achieved in reverse phase on a C18 column (5 μm particle size, 250 × 4.6 mm; Phenomenex, Torrance, CA, USA). Elution was made with sulfuric acid (3.6 mM). The compounds were identified by comparing their retention time and Ultraviolet-Visible (UV-Vis) spectra with those of standards (ascorbic, citric, and fumaric acids) and quantified based on calibration curves obtained by plotting the peak area recorded at 245 nm for ascorbic acid and at 215 nm for citric and fumaric acids against concentration. Data were recorded and processed using LabSolutions Multi Liquid Chromatography (LC)-PDA software (Shimadzu Corporation, Kyoto, Japan), and the results were given as mg per 100 g of fw and dw.

#### 2.3.4. Fatty Acids

The fatty acids obtained by Soxhlet extraction were methylated with methanol/sulfuric acid/toluene 2:1:1 (*v/v/v*) during ~12 h in a water bath at 50 °C and 160 rpm. Then, deionized water was added to obtain phase separation and the fatty acid methyl esters (FAME) were recovered with diethyl ether, dehydrated, and filtered through 0.2 μm nylon filters. The analysis was performed in a DANI gas chromatograph GC 1000 (DANI instruments, Contone, Switzerland) equipped with a split/splitless injector and a flame ionization detector (FID) at 260 °C. Chromatographic separation was performed on a Zebron-Kame column (30 m × 0.25 mm i.d., 0.20 µm film thickness, Phenomenex, Torrance, CA, USA). The operating conditions were previously described by Iyda et al. [28]. The identification was made by a chromatographic comparison of the retention times of the sample FAME peaks with those of the standard 47885-U. Data were recorded and processed using the Clarity 4.0 Software (DataApex, Podohradska, Czech Republic) and given as relative percentage (%) of each fatty acid.

#### 2.3.5. Tocopherols

Tocopherols were analyzed in the Knauer HPLC system referred above coupled with a fluorescence detector (FP-2020, Jasco, Easton, MD, USA) programmed for excitation at 290 nm and emission at 330 nm, as previously described by Pinela et al. [26]. Briefly, the sample (~500 mg) was spiked with BHT solution (10 mg/mL) and tocol (internal standard, 50 μg/mL), and homogenized with 4 mL of methanol by shaking for 1 min and then with 4 mL of hexane. Then, 2 mL of saturated NaCl solution was added, the mixture was homogenized and centrifuged at 4000× *g* for 5 min, and the upper layer was collected. The extraction was repeated twice with hexane. The obtained extracts were dried under a nitrogen stream, redissolved in 2 mL of *n*-hexane, dehydrated, and filtered through a 0.22 μm disposable syringe filter. Chromatographic separation was performed in normal phase on a Polyamide II column (5 μm particle size, 250 × 4.6 mm; YMC, Kyoto, Japan). Elution was made with a mixture of *n*-hexane and ethyl acetate (70:30, *v/v*). The detected compounds were identified by chromatographic comparisons with authentic standards (α, β, γ, and δ isoforms) and quantified using the internal standard method. Data were recorded and processed using the Clarity 2.4 software and the results were given as µg per 100 g of fw and dw.

### 2.4. Extract Preparation

The red raspberry sample (~1 g) underwent a solid-liquid extraction twice with 30 mL of an ethanol/water mixture (80:20, *v/v*) acidified with citric acid (until pH 3) for 1 h at room temperature and the supernatant was filtered through Whatman no. 4 filter paper. Ethanol was separated from the filtrate in a rotary evaporator with a bath temperature of 40 °C and the aqueous phase was lyophilized.

### 2.5. Analysis of Anthocyanins

The red raspberry extract (5 mL at 100 mg/mL H_2_O) was passed through a Sep-Pak C18 3 cc Vac cartridge (Phenomenex, Torrance, CA, USA) previously activated with 5 mL of methanol and, subsequently, with 5 mL of water. Sugars and other polar molecules were removed by passing 15 mL of water, and the anthocyanins were eluted with 5 mL of methanol. The methanolic extract was concentrated under reduced pressure, redissolved in 1 mL of methanol/water (20:80, *v/v*), and filtered through a 0.22 µm syringe filter disc. The analysis was performed by HPLC (Dionex Ultimate 3000 UPLC, Thermo Scientific, San Jose, CA, USA) coupled with a diode-array detector (DAD) and a Linear Ion Trap (LTQ XL) mass spectrometer (MS, Thermo Finnigan, San Jose, CA, USA) equipped with an electrospray ionization (ESI) source. Chromatographic separation was achieved in an AQUA^®^ (Phenomenex) reverse phase C18 column (5 μm, 150 mm × 4.6 mm). Elution was performed with 0.1% (*v/v*) TFA in water and acetonitrile [29]. The anthocyanins identification was performed in double online detection using a DAD (at a wavelength of 520 nm) coupled with the MS working in positive mode. Identification was performed comparing the fragmentation pattern, retention times, and UV–vis spectra with those of authentic standards or data available in the literature. Quantification was achieved with a seven-level calibration curve (*y* = 134578x – 3E^+06^; *r^2^* = 0.9986; limit of detection (LOD) = 0.25 µg/mL; limit of quantification (LOQ) = 0.83 µg/mL) constructed with cyanidin–3–*O*–glucoside [20–200 µg/mL]. Data were recorded and processed using Xcalibur data system (Thermo Finnigan, San Jose, CA, USA) and the results were expressed in mg per g of extract.

### 2.6. Evaluation of Bioactive Properties

#### 2.6.1. Antioxidant Activity

Oxidative hemolysis inhibition (OxHLIA), thiobarbituric acid reactive substances formation inhibition (TBARS), and β-carotene bleaching inhibition (β-CBI) assays were performed to assess the antioxidant activity of the red raspberry extract. Trolox was used as a positive control.

OxHLIA assay. An erythrocyte solution (2.8%, *v/v*; 200 µL in phosphate-buffered saline (PBS)) was mixed with 400 µL of either extract solution (0.0155–0.5 μg/mL in PBS), PBS (control), or water (for complete hemolysis). After pre-incubation at 37 °C for 10 min with shaking, 200 μL of AAPH (160 mM in PBS) was added and the optical density at 690 nm was measured every ~10 min in a microplate reader (Bio-Tek Instruments, ELX800) until complete hemolysis [30]. The delay time (Δ*t*) values (min) resulting from the half hemolysis time (H*t*_50_ values) obtained from the hemolytic curves of each extract concentration minus the H*t*_50_ value of the PBS control were correlated with the respective extract concentration to obtain IC_50_ values (µg/mL), which were calculated for a Δ*t* of 60 min.

TBARS assay. A porcine brain cell solution (1:2, *w/v*; 0.1 mL) was incubated with 0.2 mL of extract solutions (0.1563–2.5 mg/mL in water) plus 0.1 mL of FeSO_4_ (10 µM) and of ascorbic acid (0.1 mM) at 37 °C for 1 h. Then, 0.5 mL of trichloroacetic (28% *w/v*) and 0.38 mL of thiobarbituric (TBA, 2%, *w/v*) acids were added and the mixture was heated at 80 °C for 20 min. After centrifugation at 3000× *g* for 10 min, the malondialdehyde (MDA)-2TBA complexes formed in the supernatant were monitored at 532 nm (Specord 200 spectrophotometer, Analytik Jena, Jena, Germany) [31]. The results were expressed as EC_50_ values (µg/mL).

β-CBI assay. A β-carotene-linoleic acid emulsion (4.8 mL) was mixed with the extract solutions (0.0098–5 mg/mL in water; 0.2 mL) and the absorbance was measured at 470 nm as soon as mixed (A_βT0_) and after 2 h of incubation at 50 °C (A_βT0_) [31]. The β-CBI capacity was calculated as follows: (A_βT2_/A_βT0_) × 100. The results were expressed as EC_50_ values (µg/mL).

#### 2.6.2. Antimicrobial Activity

The antibacterial activity was tested against the Gram-positive *Bacillus cereus* (food isolate) and *Listeria monocytogenes* (national collection of type culture (NCTC) 7973) and the Gram-negative *Escherichia coli* (American type culture collection (ATCC) 25922) and *Salmonella typhimurium* (ATCC 13311) [32]. The activity against *Aspergillus fumigatus* (ATCC 9197), *Aspergillus niger* (ATCC 6275), *Penicillium verrucosum* var. *cyclopium* (food isolate), and *Trichoderma viride* (IAM-Culture Collection, Center for Cellular and Molecular Research, Institute of Molecular and Cellular Biosciences, University of Tokyo, Japan, 5061) was also tested [33]. The microorganisms were obtained from the Mycological laboratory, Department of Plant Physiology, Institute for biological research “Sinisa Stanković”, University of Belgrade, Serbia. The minimum inhibitory concentrations (MIC) were determined by the serial microdilution method and the rapid *p*-iodonitrotetrazolium violet (INT) colorimetric assay as previously described [32,33]. MICs were defined as the lowest extract concentration (mg/mL) that inhibits the visible microbial growth (at the binocular microscope). The minimal bactericidal and fungicidal concentrations (MBC and MFC, respectively) were determined by measuring the lowest concentration (mg/mL) that yielded no growth; therefore, MBC and MFC were the lowest extract concentration required to kill the original inoculum. Sodium benzoate (E211) and potassium metabisulfite (E224) were used as positive controls, while 5% dimethyl sulfoxide (DMSO) was used as a negative control.

### 2.7. Statistical Analysis

All the analyses were performed in triplicate and the results were presented as mean ± standard deviation (except for the antimicrobial activity). For the antioxidant activity, the differences between the extract and control were assessed by applying a two-tailed paired Student’s *t*-test. All the statistical tests were performed at a 5% significance level using SPSS Statistics software (IBM SPSS Statistics for Windows, Version 22.0, IBM Corp., Armonk, NY, USA).

## 3. Results and Discussion

### 3.1. Nutritional Composition

Red raspberry is commonly consumed fresh and also in dried form in cereals, trail mixes, and chocolates, among other food products. Therefore, the results of its nutritional composition are presented in both fresh and dry weight (Table 1). About 80% of this fruit consisted of water. Carbohydrates were the most abundant macronutrients, with a 100 g portion containing 16.12 ± 0.01 g. These constituents are also predominant in cherry (*Prunus avium* L., 11.94 g/100 g), blueberry (*Vaccinium corymbosum* L., 11.54 g/100 g), blackberry (*Rubus* spp., 10.18 g/100 g), and strawberry (*Fragaria* × *ananassa* Duch., 6.30 g/100 g) [10]. Ashes ranked second with 0.66 ± 0.02 g/100 g, followed by proteins with 0.18 ± 0.01 g/100 g and fat with only 0.132 ± 0.005 g/100 g. These values are lower than those previously reported by De Souza et al. [10] for moisture (88.6 g/100 g) and fat (0.28 g/100 g) and higher than those found for ash (0.25 g/100 g) and proteins (1.00 g/100 g). The Portuguese Food Information Resource (PortFIR) platform [34] presents higher amounts of proteins (0.9 g), fat (0.6 g), and moisture (84.3 g) for 100 g edible portions of fresh raspberry, and lower values of carbohydrates (5.1 g) and ash (0.54 g). All these differences can be explained by the edaphoclimatic conditions of the cultivation sites, the agricultural practices adopted, the genetic characteristics, and the cultivar under study [11]. The fat content can also vary depending on the quantity of seeds present in the fruit [35].

In terms of energy, each 100 g serving provides only 66.40 ± 0.04 kcal (Table 1), a value higher than that calculated by De Souza et al. [10] (46 ± 0.9 kcal) and that found in the PortFIR database (49 kcal) [34], but closer to that found in the United States Department of Agriculture (USDA) database (52 kcal) [36]. Therefore, “Kweli” red raspberry appears as a suitable food for low-fat and low-calorie diets.

The fruit flavor and taste are highly influenced by the sugar concentration. Table 1 shows the free sugars composition of the studied red raspberry. Two monosaccharides (fructose and glucose), two disaccharides (sucrose and trehalose), and one trisaccharide (raffinose) were detected. Among these, fructose was the major saccharide (2.42 ± 0.03 g/100 g), followed by glucose (2.13 ± 0.09 g/100 g), sucrose (1.41 ± 0.02 g/100 g), and finally trehalose (0.020 ± 0.001 g/100 g) in a smaller amount. A 100 g edible portion contained 6.0 ± 0.1 g of free sugars. These results are in agreement with those previously reported by Kafkas et al. [12], which quantified 2.5–3.5 g of fructose, 2.1–2.8 g of glucose, 0.7–1.6 g of sucrose, and 5.8–7.9 g of total free sugars in 100 g servings of seven red raspberry cultivars (“Canby”, “Tulameen”, “Willamette”, “Hollanda Boduru”, “Heritage”, “Meeker”, and “Newburg”) grown in an experimental field in Turkey. The total free sugars content was also comparable with the ~5.6 g/100 g reported by Stojanov et al. [11] for the “Meeker” floricane-fruiting red raspberry cultivar grown in western Serbia. According to Milivojević et al. [37], the sugar content in red raspberry and other *Rubus* species can vary between cultivars and wild relatives. The authors found fructose as the major free sugar in the “Willamette” and “Meeker” cultivars (4.7–4.9 g/100 g fw), followed by glucose (3.5–3.6 g/100 g fw) and sucrose (0.53–0.64 g/100 g fw), but the quantified levels were higher for both monosaccharides than those herein reported for the “Kweli” cultivar (Table 1). Thus, 100 g portions of ripe “Willamette” and “Meeker” raspberries contained 8.84 and 9.07 g/100 g of total free sugars. In turn, Milivojević and co-workers [37] reported a slightly lower free sugars content (7.37 g/100 g fw) in red raspberries harvested from native populations in Western Serbia.

A higher sugar concentration does not always result in sweeter–tasting raspberries, since the organic acid levels also contribute to the taste perception. In Table 1, it is possible to observe that citric acid was the most abundant organic acid detected, reaching 2.7 ± 0.1 g/100 g of fresh red raspberry, in accordance with previously reports (1.6–2.8 g/100 g fw) [1]. Citric acid was also identified as the major organic acid (mean value, 1.31 g/100 g fw) in wild red raspberry accessions from northern Turkey, in which small amounts of malic and ascorbic acids were also detected [13], as well as in samples from the south of Minas Gerais, Brazil (1.88 g citric acid/100 g fw) [10], and those harvested at experimental fields in Leikanger, Western Norway (1.75–2.23 g citric acid/100 g fw) [14]. According to De Souza et al. [10], red raspberry has a higher acidity than other berries such as blackberry, strawberry, blueberry, and cherry.

In this study, ascorbic acid was detected and quantified in the “Kweli” red raspberry (Table 1), with a concentration (17 ± 1 mg/100 g fw) within the values described for 17 cultivars grown in Lithuania (16.4–24.4 mg/100 g fw) [38], 5 cultivars gown in Western Norway (17–47 mg/100 g fw) [14], 4 cultivars planted in South-Eastern Norway (15.4–32.0 mg/100 g fw) [15], and the “Erika” cultivar produced in Pergine Val-sugana, Italy (17–20 mg/100 g fw) [16]. Therefore, a 100-g serving of fresh “Kweli” raspberries provides 18.9% and 22.7% of the recommended dietary allowance (RDA) of vitamin C for healthy adult men and women, respectively, while a 100 g serving of dried raspberries exceeds the RDA for this water-soluble vitamin [39]. A significantly higher ascorbic acid level (reaching 92 ± 10 mg/100 g fw) has already been described for red raspberry [10], but a colorimetric method with 2,4-dinitrophenylhydrazine was used for quantification, which may be associated with an overestimation of this vitamin.

Acidity helps to provide the desirable sugar/acid balance, which is important for the pleasant raspberry taste and thus for consumer acceptance. The sweetness index of 2.2 herein obtained for the “Kweli” red raspberry (calculated as the total sugars/total organic acids ratio) was lower than that previously described by Stojanov et al. [11] for the “Meeker” cultivar (2.64), mainly due to the lower titratable acidity recorded by the authors.

The results of the tocopherols composition of red raspberry are also shown in Table 1. The isoforms α-, γ- and δ- were detected, and δ-tocopherol was the most abundant (1.36 ± 0.03 mg/100 g), followed by γ-tocopherol (0.52 ± 0.02 mg/100 g) and, to a lesser extent, α-tocopherol (0.050 ± 0.001 mg/100 g). The total tocopherols content in the studied raspberry cultivar was 1.92 ± 0.05 mg/100 g fw and 11.4 ± 0.3 mg/100 g dw. Large variations in the tocopherol levels between cultivar have been reported in ripe red raspberries. Carvalho et al. [40] described γ-tocopherol (11.5–12.2 mg/100 g dw) as the major vitamer in the red varieties “Tulameen” and ‘Sugana’, followed by δ-tocopherol (5.2–7.7 mg/100 g dw) and α-tocopherol (0.75–2.1 mg/100 g dw), and a total tocopherols content ranging from 17.4 to 22.0 mg/100 g dw. In another study, Miret and Munné-Bosch [41] detected the four tocopherol homologues in “Heritage” raspberries from a nursery in Ejea de los Caballeros, Spain, and descried α- and δ-tocopherols as the major forms. The authors also reported the absence of tocotrienols. Parry et al. [42] reported α-, γ-, and δ-tocopherols in cold-pressed red raspberry seed oil, with the predominance of the γ- isoform. For dietary purposes, the vitamin E activity has been expressed as α-tocopherol equivalents, but all isoforms display antioxidant activity and health promoting effects [43].

**Figure 1:**
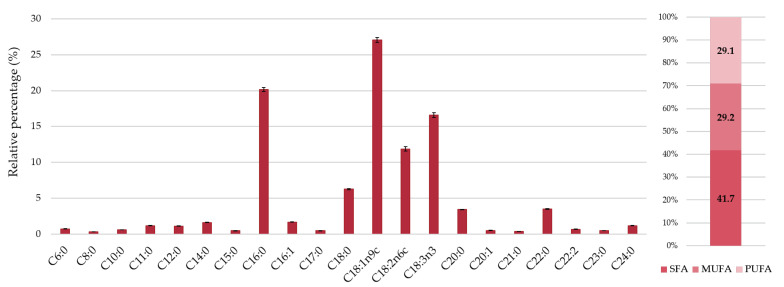
Fatty acids composition of the “Kweli” red raspberry and the relative percentage of saturated (SFA), monounsaturated (MUFA), and polyunsaturated (PUFA) fatty acids.

In addition to tocopherols, other liposoluble compounds such as fatty acids have been analyzed in red raspberry. The fatty acid profile of the “Kweli” cultivar is shown in Figure 1 and Table S1. Twenty-one fatty acids were identified and the most abundant were oleic (C18:1*n*-9c, 27.1%), palmitic (C16:0, 20.2%), α-linolenic (C18:3*n*-3, 16.6%), and linoleic (C18:2*n*-6c, 11.9%) acids. Saturated fatty acids (SFA) were detected in large quantities (41.7%), mainly due to the high levels of C16:0 already mentioned, but also stearic (C18:0, 6.25%), arachidic (C20:0, 3.41%), and behenic (C22:0, 3.48%) acids. In turn, monounsaturated (MUFA) and polyunsaturated (PUFA) fatty acids were detected in an equal proportion (~29%). Thus, it was possible to conclude that about 58.3% of the raspberry’s lipid fraction consists of unsaturated fatty acids. Nine of the detected fatty acids were previously reported by Kafkas et al. [12] in red raspberry cultivars grown in Turkey, which contained SFA, MUFA, and PUFA levels ranging from 4.97% to 20.31%, 14.65% to 18.47%, and 62.85% to 78.68%, respectively. Therefore, the proportion of fatty acids varied widely between the cultivars. In another study, Parry et al. [42] also identified C18:2*n*-6, C18:3*n*-3, and C18:1 as the major fatty acids in red raspberry seed oil. Comparing with hill raspberry (*Rubus niveus* Thunb.), Caidan et al. [44] described a different profile consisting mainly of palmitic (36.8%), linolenic (30.1%), linoleic (29.3%), and stearic (5.4%) acids. Edaphoclimatic and genetic factors may justify the observed variations. For example, Ferreyra et al. [45] concluded that avocado fruit grown in areas with higher temperatures had lower amounts of C18 fatty acids and higher levels of C16 fatty acids.

The PUFA/SFA and PUFA *n*-6/*n*-3 ratios have been used as important indicators of the nutritional quality and healthiness of food products, and these ratios should be over 0.45 and lower than 4.0, respectively [46]. As presented in Table S1, the studied red raspberry cultivar meets the requirements mentioned for these indices (PUFA/SFA ratio of 0.7 and PUFA *n*-3/*n*-6 ratio of 1.4), which highlights its nutritional value, even though it has a quite low crude fat content (Table 1).

### 3.2. Anthocyanins Composition

**Figure 2:**
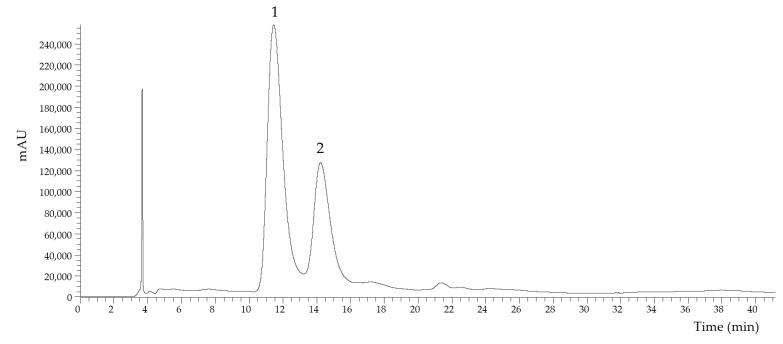
HPLC anthocyanin profile of the extract of “Kweli” red raspberry recorded at 520 nm. Peak identification is presented in Table 2.

Berries in general have been described as interesting sources of polyphenols, particularly anthocyanins [10,21]. The anthocyanins profile of the “Kweli” red raspberry (shown in Figure 2) was characterized by HPLC–DAD–ESI/MS*^n^*, and the results are presented in Table 2. Two anthocyanins (cyanidin–*O*–sophoroside and cyanidin–*O*–hexoside) were identified based on their retention time, UV–Vis, and mass spectra, and the data are available in the literature [19,20]. Compound number 1 presented a pseudomolecular ion [M − H]^+^ at *m/z* 611 and an MS^2^ fragment at *m/z* 287, being identified as cyanidin–*O*–sophoroside [19,20]. Compound number 2 was identified as cyanidin–*O*–hexoside due to the pseudomolecular ion [M − H]^+^ at *m/z* 449 that yielded a fragment at *m/z* 287 [19,20]. Cyanidin–*O*–sophoroside was the predominant anthocyanin, with 2.82 ± 0.03 mg/g of extract (Table 2).

Previous studies described glycosylated cyanidins as 98% of the red raspberry anthocyanins and pelargonidins corresponding to 2% [17]. Cyanidin 3–*O*–sophoroside has also been described as the most abundant anthocyanin in the “Ljulin” red raspberries obtained in Skierniewice, Poland, followed by cyanidin 3–*O*–glucoside and pelargonidin 3–*O*–sophoroside. In this cultivar, the pelargonidin glycosides corresponded to 7% of the total content of anthocyanins [18]. However, no pelargonidins were detected in our sample, nor in the “Kweli” and “Tulameen” cultivars grown under organic agricultural practices in Braga, Portugal [21], or in the “Veten” red raspberry cultivated in Skierniewice, Poland [18]. Moreover, the main anthocyanins in raspberries may differ according to the species. Stavang et al. [47] described cyanidin–3–sophoroside and cyanidin–3–glucoside in red raspberry, and Kula et al. [48] identified cyanidin–3–rutinoside in black raspberry (*Rubus occidentalis*).

The deep red coloration of red raspberry is related to its anthocyanin composition, whose concentration is influenced by several factors, such as the variety, cultivar, maturation stage, and edaphoclimatic characteristics of the growing sites [49], and anthocyanins have been important targets for breeding efforts to improve the consumers’ perception of quality. Sun exposure increases the concentration of these water-soluble vacuolar pigments, as it promotes the expression of flavonoid biosynthetic genes in the skin of these fruits. On the other hand, high temperatures lead to the suppression of the biosynthesis of these pigments [50].

### 3.3. Bioactive Properties

The consumer preference for fruits and vegetables with greater bioactive effects has grown in recent years. In this study, the antioxidant activity of the hydroethanolic extract obtained with the “Kweli” red raspberry cultivar was evaluated in vitro for its ability to: (i) prevent lipid peroxidation by inhibiting the formation of thiobarbituric acid substances reactive (TBARS) using porcine brain tissues as a biological substrate, (ii) to inhibit the oxidative hemolysis (OxHLIA) of erythrocytes isolated from sheep’s blood, and (iii) to prevent the discoloration/bleaching of β-carotene. The results are shown in Table 3 and expressed as IC_50_ or EC_50_ values (µg/mL). Thus, the lower these values, the greater the antioxidant activity of the red raspberry extract.

In the TBARS assay, an EC_50_ of 122 ± 2 µg/mL was obtained with the red raspberry extract, a value much higher than that of the trolox (5.4 ± 0.3 µg/mL) used as a positive control (Table 3), which demonstrates the lower antioxidant effect of the natural extract compared to this commercial antioxidant. Nevertheless, it should be noted that plant extracts are complex mixtures of different compounds (e.g., polyphenols, vitamins, carbohydrates, etc.) with or without antioxidant capacity, while trolox is a pure antioxidant compound. This assay made it possible to assess whether the red raspberry extract can prevent the formation of reactive substances, such as malondialdehyde (MDA), that result from the oxidation of PUFA present in the porcine brain cell membranes. When the extract fails to prevent the formation of TBARS such as MDA, it reacts with thiobarbituric acid (TBA) that is added to the mixture before incubation at 80 °C to form pink-colored MDA-TBA2 complexes that absorb at a wavelength 532 nm.

The red raspberry extract gave an EC_50_ of 18.7 ± 0.2 µg/mL in the β-carotene bleaching inhibition assay (β-CBI), which was higher than that of the positive control (0.20 ± 0.02 µg/mL) (Table 3). The obtained value translates the extract concentration necessary to inhibit half of the oxidation (discoloration) of β-carotene, which is attacked in vitro by linoleate radicals that are derived from the degradation of the linoleic acid added to the emulsion. The presence of natural antioxidants protects this red-orange lipophilic pigment from the free-radical attack, thus preserving the characteristic orange color of β-carotene.

The IC_50_ values obtained in the OxHLIA assay translate the extract concentration required to protect half of the erythrocyte population from oxidative hemolysis during a Δ*t* of 60 min. As observed in Table 3, 298 ± 13 µg/mL of red raspberry extract was required, while 19 ± 1 µg/mL of trolox was enough. In this assay, the temperature-dependent free radical initiator AAPH is responsible for the formation of free (peroxyl) radicals in the in vitro system that, in a first instance, attack the erythrocyte membranes and eventually cause hemolysis. Then, lipophilic radicals are generated as a consequence of this initial lipid peroxidation phenomenon. Since the peroxyl radicals formed in the in vitro system are also found in the human body, this cell-based assay has been pointed out as suitable for assessing the antioxidant activity of natural extracts.

The antioxidant activity of red raspberry has been evaluated mainly for its ability to eliminate free radicals such as DPPH and ABTS, for its ferric (FRAP) and cupric (CUPRAC) reducing antioxidant potential, and also for its ability to prevent β-carotene discoloration [5,10,19,37]. Some authors have already reported that the hydromethanolic extract of red raspberry is more antioxidant than that of blueberry, which had lower levels of phenolic compounds such as anthocyanins [10]. It is worth noticing that most of the existing antioxidant activity studies employed assays with no correlation with any biological system. The results obtained in this study with OxHLIA are of grader biological relevance when compared to those obtained with chemical-based methods for the reasons mentioned above. Moreover, erythrocytes are metabolically simplified model system since they have intrinsically poor repair mechanisms and, in the human body, are typically exposed to oxidative stressors owing to their specific role as oxygen carriers in the blood [51]. In a previous study, Gião et al. [51] tested the ability of an aqueous raspberry extract to inhibit the oxidative hemolysis but using human erythrocytes and hydrogen peroxide (a normal cell metabolite) as oxidizing agent. In an in vivo study, Noratto et al. [22] fed obese diabetic mice with red raspberry (isocaloric diet with 5.3% lyophilized raspberries, *w/w*) and observed a decrease in oxidative stress biomarkers. The authors also linked the enhanced detoxifying cell defenses exerted by the red raspberry intake to its polyphenols and dietary fiber.

The minimum inhibitory (MIC) and bactericidal (MBC) or fungicidal (MFC) concentrations obtained with the raspberry extract against the four foodborne bacteria and the four fungi using the serial microdilution method and the rapid INT colorimetric assay are shown in Table 3. The study showed the antibacterial potential of the red raspberry extract against all the tested strains, with MIC values ranging from 0.78 to 3.12 mg/mL and MBC values from 1.56 to 6.24 mg/mL for *Bacillus cereus* and the remaining bacteria, respectively. Thus, *Bacillus cereus* was the most susceptible bacterium to the red raspberry extract, which was more effective than potassium metabisulfite (a food additive known as E224) in inhibiting (2 mg/mL) and killing (4 mg/mL) this Gram-positive spore-forming bacterium commonly found in food products. In an earlier study, Krauze-Baranowska et al. [18] screened the antimicrobial activity of hydroethanolic extracts of red and black raspberries and found that both extracts were active against the studied Gram-positive and Gram-negative bacteria. The bacterial sensitivity to the natural extracts varied widely, and the most sensitive were *Corynebacterium diphtheriae* and *Moraxella catarrhalis*. This sensitivity was attributed to sanguiin H-6 and ellagic acid, compounds not detected in the cultivar analyzed in the present study.

For antifungal activity, values ranging from 1.56 to >6.24 mg/mL were obtained for the studied fungi (Table 3). The most promising effect was found against *Trichoderma viride*, with a MIC of 1.56 mg/mL and a MFC of 3.12 mg/mL, thus being the most susceptible microorganism to the red raspberry extract. This was followed by the fungus *Aspergillus niger*, with a MIC of 3.12 mg/mL and a MFC of 6.24 mg/mL, which is one of the most common species of the genus *Aspergillus* and a common food contaminant. The fungi *Aspergillus fumigatus* and *Penicillium verrucosum* var. *cyclopium* showed equal MIC and MFC values (>6.24 mg/mL). The food additives (E211 and E224) used as positive controls gave values between 0.5 and 4 mg/mL, thus being more effective than the red raspberry extract. The antifungal activity of ellagitannins isolated from red raspberry against *Geotrichum candidum* was already demonstrated by Klewicka et al. [52] both in vitro and in situ.

## 4. Conclusions

The dehydrated “Kweli” red raspberry was characterized as having interesting levels of total carbohydrates, where fructose and glucose were the major free sugars, and also of proteins and ashes. The fat content was quite low and constituted mainly unsaturated fatty acids (58.3%), with a predominance of oleic, α-linolenic, and linoleic acids. The nutritional quality and healthiness of “Kweli” were also recognized by PUFA/SFA and *n*-6/*n*-3 ratios over 0.45 and below 4, respectively. Hundred-gram servings of fresh red raspberry contain high levels of citric and ascorbic acids, and δ-tocopherol. The anthocyanins cyanidin-*O*-hexoside and cyanidin-*O*-sophoroside were identified in the red raspberry extract, which was able to inhibit TBARS formation, oxidative hemolysis, and β-carotene bleaching. *Bacillus cereus* was the most susceptible bacterium to the extract, which was more effective than the food additive E224, and *Trichoderma viride* was the most susceptible fungus. All these results highlighted the nutritional quality of the “Kweli” cultivar and showed that its composition differs in some parameters from the other red raspberry cultivars already characterized.

In future studies, it will be interesting to monitor and track red raspberry contaminants to ensure their safety and to explore the coloring potential of red raspberry anthocyanins by developing cyanidin-rich ingredients for their incorporation into food products as natural colorants. However, fruit waste should be used in these studies to promote a sustainable production/consumption system.

## Figures and Tables

**Table 1:** Nutritional value and free sugars, organic acids, and tocopherols composition of “Kweli” red raspberry.

Constituent	Content (fw)	Content (dw)
Moisture (g/100 g) ^1^	83.1 ± 0.2	-
Proteins (g/100 g)	0.18 ± 0.01	6.8 ± 0.4
Ash (g/100 g)	0.66 ± 0.02	3.90 ± 0.09
Fat (g/100 g)	0.132 ± 0.005	0.78 ± 0.03
Total carbohydrates (g/100 g)	16.12 ± 0.01	88.5 ± 0.3
Energy (kcal/100 g)	66.40 ± 0.04	388 ± 1
Fructose (g/100 g)	2.42 ± 0.03	14.3 ± 0.2
Glucose (g/100 g)	2.13 ± 0.09	12.6 ± 0.5
Sucrose (g/100 g)	1.41 ± 0.02	8.3 ± 0.1
Trehalose (g/100 g)	0.020 ± 0.001	0.140 ± 0.008
Raffinose (g/100 g)	0.040 ± 0.001	0.260 ± 0.002
Total sugars (g/100 g)	6.0 ± 0.1	35.7 ± 0.8
Ascorbic acid (mg/100 g)	17 ± 1	100 ± 7
Citric acid (mg/100 g)	2718 ± 134	16066 ± 790
Fumaric acid (mg/100 g)	1.5 ± 0.1	8.9 ± 0.6
Total organic acids (mg/100 g)	2765 ± 136	16175 ± 798
α-Tocopherol (mg/100 g)	0.050 ± 0.001	0.29 ± 0.01
γ-Tocopherol (mg/100 g)	0.52 ± 0.02	3.1 ± 0.01
δ-Tocopherol (mg/100 g)	1.36 ± 0.03	8.0 ± 0.2
Total tocopherols (mg/100 g)	1.92 ± 0.05	11.4 ± 0.3

^1^ The results are presented as mean ± standard deviation. fw: fresh weight; dw: dry weight.

**Table 2:** Anthocyanins identified and quantified in the extract of “Kweli” red raspberry. Here is presented the retention time (R*t*), the wavelengths of maximum absorption in the UV-vis region (λ_max_), the pseudomolecular and MS^2^ fragment ions, and the relative abundance in brackets.

Peak	R*t* (min)	λ_max_ (nm)	[M − H]^+^ (*m/z*)	MS^2^ Fragments (*m/z*)	Tentative Identification	Content (mg/g Extract)
1	11.48	515	611	287 (100)	Cyanidin-*O*-sophoroside	2.82 ± 0.03
2	14.26	512	449	287 (100)	Cyanidin-*O*-hexoside	1.69 ± 0.02
					Total anthocyanins	4.51 ± 0.04

**Table 3:** Antioxidant and antimicrobial activities of the “Kweli” red raspberry extract and positive controls.

Bioactivity	Red Raspberry Extract		Positive Controls			
Antioxidant activity			Trolox			
TBARS assay (EC_50_, µg/mL)	122 ± 2		5.4 ± 0.3			
OxHLIA assay (IC_50_, µg/mL)	298 ± 17		19 ± 1			
β-CBI assay (EC_50_, µg/mL)	18.7 ± 0.2		0.20 ± 0.02			
			E211		E224	
Antibacterial activity	MIC	MBC	MIC	MBC	MIC	MBC
*Bacillus cereus*	0.78	1.56	0.5	0.5	2	4
*Listeria monocytogenes*	3.12	6.24	1	2	0.5	1
*Escherichia coli*	3.12	6.24	1	2	0.5	1
*Salmonella typhimurium*	3.12	6.24	1	2	1	1
Antifungal activity	MIC	MFC	MIC	MFC	MIC	MFC
*Aspergillus fumigatus*	>6.24	>6.24	1	2	1	1
*Aspergillus niger*	3.12	6.24	1	2	1	1
*Penicillium verrucosum* var. *cyclopium*	>6.24	>6.24	2	4	1	1
*Trichoderma viride*	1.56	3.12	1	2	0.5	0.5

In the antioxidant activity, the mean values of the extract differed statistically from those of trolox (*p* < 0.001) when applying a Student’s *t*-test. TBARS: thiobarbituric acid reactive substances; OxHLIA: oxidative hemolysis inhibition assay; β-CBI: β-carotene bleaching inhibition; EC_50_: extract concentration providing 50% of antioxidant activity; IC_50_: extract concentration required to protect 50% of the erythrocyte population from the oxidative hemolysis for a Δ*t* of 60 min; E211: sodium benzoate; E224: potassium metabisulfite; MIC: minimum inhibitory concentration (mg/mL); MBC: minimum bactericidal concentration (mg/mL); MFC: minimum fungicidal concentration (mg/mL).

## Supplementary Materials

The following are available online at https://www.mdpi.com/2304-8158/9/11/1522/s1: Table S1: Fatty acids composition of “Kweli” red raspberry.

## References

[1] Schulz M., Chim J.F. (2019). Nutritional and bioactive value of *Rubus* berries. Food Biosci..

[2] Kim M.J., Sutton K.L., Harris G.K., Caballero B., Finglas P.M., Toldrá F. (2016). Raspberries and Related Fruits. Encyclopedia of Food and Health.

[3] FAOSTAT. http://www.fao.org/faostat/en/?#data/QC.

[4] Overview Global Berry Market—FreshPlaza. https://www.freshplaza.com/article/9136082/overview-global-berry-market/.

[5] Valdés García A., Maestre Pérez S.E., Butsko M., Prats Moya M.S., Beltrán Sanahuja A. (2020). Authentication of “Adelita” raspberry cultivar based on physical properties, antioxidant activity and volatile profile. Antioxidants.

[6] Andrianjaka-Camps Z.N., Wittemann M.S., Ançay A., Carlen C. (2016). New cultivars for quality production of primocane fruiting raspberries enriched in healthy compounds. Acta Hortic..

[7] Advanced Berry Breeding—Kweli®. https://www.abbreeding.nl/varieties/kweli/?lang=en.

[8] Strik B.C., Moore P.P. (2014). Raspberry Cultivars for the Pacific Northwest.

[9] Hanson E., Crain B., Hanson K. (2019). Response of potted red raspberry cultivars to double-cropping under high tunnels. HortScience.

[10] De Souza V.R., Pereira P.A.P., Da Silva T.L.T., De Oliveira Lima L.C., Pio R., Queiroz F. (2014). Determination of the bioactive compounds, antioxidant activity and chemical composition of Brazilian blackberry, red raspberry, strawberry, blueberry and sweet cherry fruits. Food Chem..

[11] Stojanov D., Milošević T., Mašković P., Milošević N., Glišić I., Paunović G. (2019). Influence of organic, organo-mineral and mineral fertilisers on cane traits, productivity and berry quality of red raspberry (*Rubus idaeus* L.). Sci. Hortic..

[12] Kafkas E., Özgen M., Özoğul Y., Türemiş N. (2008). Phytochemical and fatty acid profile of selected red raspberry cultivars: A comparative study. J. Food Qual..

[13] Çekiç Ç., Özgen M. (2010). Comparison of antioxidant capacity and phytochemical properties of wild and cultivated red raspberries (*Rubus idaeus* L.). J. Food Compos. Anal..

[14] Mazur S.P., Nes A., Wold A.B., Remberg S.F., Aaby K. (2014). Quality and chemical composition of ten red raspberry (*Rubus idaeus* L.) genotypes during three harvest seasons. Food Chem..

[15] Haffner K., Rosenfeld H.J., Skrede G., Wang L. (2002). Quality of red raspberry *Rubus idaeus* L. cultivars after storage in controlled and normal atmospheres. Postharvest Biol. Technol..

[16] Giovanelli G., Limbo S., Buratti S. (2014). Effects of new packaging solutions on physico-chemical, nutritional and aromatic characteristics of red raspberries (*Rubus idaeus* L.) in postharvest storage. Postharvest Biol. Technol..

[17] Bowen-Forbes C.S., Zhang Y., Nair M.G. (2010). Anthocyanin content, antioxidant, anti-inflammatory and anticancer properties of blackberry and raspberry fruits. J. Food Compos. Anal..

[18] Krauze-Baranowska M., Majdan M., Hałasa R., Głód D., Kula M., Fecka I., Orzeł A. (2014). The antimicrobial activity of fruits from some cultivar varieties of *Rubus idaeus* and *Rubus occidentalis*. Food Funct..

[19] Sariburun E., Şahin S., Demir C., Türkben C., Uylaşer V. (2010). Phenolic content and antioxidant activity of raspberry and blackberry cultivars. J. Food Sci..

[20] Beekwilder J., Jonker H., Meesters P., Hall R.D., Van Der Meer I.M., De Vos C.H.R. (2005). Antioxidants in raspberry: On-line analysis links antioxidant activity to a diversity of individual metabolites. J. Agric. Food Chem..

[21] Anjos R., Cosme F., Gonçalves A., Nunes F.M., Vilela A., Pinto T. (2020). Effect of agricultural practices, conventional vs organic, on the phytochemical composition of ‘Kweli’ and ‘Tulameen’ raspberries (*Rubus idaeus* L.). Food Chem..

[22] Noratto G.D., Chew B.P., Atienza L.M. (2017). Red raspberry (*Rubus idaeus* L.) intake decreases oxidative stress in obese diabetic (db/db) mice. Food Chem..

[23] Kowalska K., Olejnik A., Zielińska-Wasielica J., Olkowicz M. (2019). Raspberry (*Rubus idaeus* L.) fruit extract decreases oxidation markers, improves lipid metabolism and reduces adipose tissue inflammation in hypertrophied 3T3-L1 adipocytes. J. Funct. Foods.

[24] Latimer G.W., AOAC International (2016). Official Methods of Analysis of AOAC International.

[25] European Union (2011). Regulation (EU) No 1169/2011 of the European Parliament and of the Council of 25 October 2011. Off. J. Eur. Union.

[26] Pinela J., Barreira J.C.M., Barros L., Cabo Verde S., Antonio A.L., Carvalho A.M., Oliveira M.B.P.P., Ferreira I.C.F.R. (2016). Suitability of gamma irradiation for preserving fresh-cut watercress quality during cold storage. Food Chem..

[27] Pereira C., Barros L., Carvalho A.M., Ferreira I.C.F.R. (2013). Use of UFLC-PDA for the analysis of organic acids in thirty-five species of food and medicinal plants. Food Anal. Methods.

[28] Iyda J.H., Fernandes Â., Ferreira F.D., Alves M.J., Pires T.C.S.P., Barros L., Amaral J.S., Ferreira I.C.F.R. (2019). Chemical composition and bioactive properties of the wild edible plant *Raphanus raphanistrum* L.. Food Res. Int..

[29] Gonçalves G.A., Soares A.A., Correa R.C.G., Barros L., Haminiuk C.W.I., Peralta R.M., Ferreira I.C.F.R., Bracht A. (2017). Merlot grape pomace hydroalcoholic extract improves the oxidative and inflammatory states of rats with adjuvant-induced arthritis. J. Funct. Foods.

[30] Lockowandt L., Pinela J., Roriz C.L., Pereira C., Abreu R.M.V., Calhelha R.C., Alves M.J., Barros L., Bredol M., Ferreira I.C.F.R. (2019). Chemical features and bioactivities of cornflower (*Centaurea cyanus* L.) capitula: The blue flowers and the unexplored non-edible part. Ind. Crops Prod..

[31] Pinela J., Antonio A.L., Barros L., Barreira J.C.M., Carvalho A.M., Oliveira M.B.P.P., Santos-Buelga C., Ferreira I.C.F.R. (2015). Combined effects of gamma-irradiation and preparation method on antioxidant activity and phenolic composition of *Tuberaria lignosa*. RSC Adv..

[32] Soković M., Glamočlija J., Marin P.D., Brkić D., van Griensven L.J.L.D. (2010). Antibacterial effects of the essential oils of commonly consumed medicinal herbs using an in vitro model. Molecules.

[33] Soković M., van Griensven L.J.L.D. (2006). Antimicrobial activity of essential oils and their components against the three major pathogens of the cultivated button mushroom, *Agaricus bisporus*. Eur. J. Plant Pathol..

[34] INSA PortFIR—Plataforma Portuguesa de Informação Alimentar. http://portfir.insa.pt/foodcomp/food?14110.

[35] Van Hoed V., De Clercq N., Echim C., Andjelkovic M., Leber E., Dewettinck K., VerhÉ R. (2009). Berry seeds: A source of specialty oils with high content of bioactives and nutritional value. J. Food Lipids.

[36] USDA FoodData Central—Raspberries, Red, Raw. https://fdc.nal.usda.gov/fdc-app.html#/food-details/786783/nutrients.

[37] Milivojević J., Maksimović V., Nikolić M., Bogdanović J., Maletić R., Milatović D. (2011). Chemical and antioxidant properties of cultivated and wild fragaria and *Rubus* berries. J. Food Qual..

[38] Bobinait R., Viškelis P., Venskutonis P.R. (2012). Variation of total phenolics, anthocyanins, ellagic acid and radical scavenging capacity in various raspberry (*Rubus* spp.) cultivars. Food Chem..

[39] IOM (Institute of Medicine) (2000). Dietary Reference Intakes for Vitamin C, Vitamin E, Selenium, and Carotenoids.

[40] Carvalho E., Fraser P.D., Martens S. (2013). Carotenoids and tocopherols in yellow and red raspberries. Food Chem..

[41] Miret J.A., Munné-Bosch S. (2016). Abscisic acid and pyrabactin improve vitamin C contents in raspberries. Food Chem..

[42] Parry J., Su L., Luther M., Zhou K., Peter Yurawecz M., Whittaker P., Yu L. (2005). Fatty acid composition and antioxidant properties of cold-pressed marionberry, boysenberry, red raspberry, and blueberry seed oils. J. Agric. Food Chem..

[43] Wells S.R., Jennings M.H., Rome C., Hadjivassiliou V., Papas K.A., Alexander J.S. (2010). α-, γ- and δ-tocopherols reduce inflammatory angiogenesis in human microvascular endothelial cells. J. Nutr. Biochem..

[44] Caidan R., Cairang L., Liubin, Yourui S. (2013). Simultaneous analysis of fatty acids in *Rubus niveus* Thunb. fruits by HPLC-MS/MS. Asian J. Chem..

[45] Ferreyra R., Sellés G., Saavedra J., Ortiz J., Zúñiga C., Troncoso C., Rivera S.A., González-Agüero M., Defilippi B.G. (2016). Identification of pre-harvest factors that affect fatty acid profiles of avocado fruit (*Persea americana* Mill) cv. “Hass” at harvest. S. Afr. J. Bot..

[46] Mapiye C., Chimonyo M., Dzama K., Hugo A., Strydom P.E., Muchenje V. (2011). Fatty acid composition of beef from Nguni steers supplemented with *Acacia karroo* leaf-meal. J. Food Compos. Anal..

[47] Stavang J.A., Freitag S., Foito A., Verrall S., Heide O.M., Stewart D., Sønsteby A. (2015). Raspberry fruit quality changes during ripening and storage as assessed by colour, sensory evaluation and chemical analyses. Sci. Hortic..

[48] Kula M., Majdan M., Głód D., Krauze-Baranowska M. (2016). Phenolic composition of fruits from different cultivars of red and black raspberries grown in Poland. J. Food Compos. Anal..

[49] Lee J., Dossett M., Finn C.E. (2012). *Rubus* fruit phenolic research: The good, the bad, and the confusing. Food Chem..

[50] Di Vittori L., Mazzoni L., Battino M., Mezzetti B. (2018). Pre-harvest factors influencing the quality of berries. Sci. Hortic..

[51] Gião M.S., Leitão I., Pereira A., Borges A.B., Guedes C.J., Fernandes J.C., Belo L., Santos-Silva A., Hogg T.A., Pintado M.E. (2010). Plant aqueous extracts: Antioxidant capacity via haemolysis and bacteriophage P22 protection. Food Control.

[52] Klewicka E., Sójka M., Klewicki R., Kołodziejczyk K., Lipińska L., Nowak A. (2016). Ellagitannins from raspberry (*Rubus idaeus* L.) fruit as natural inhibitors of *Geotrichum candidum*. Molecules.

