# Supplementary material for: Compositional Features of the “Kweli” Red Raspberry and Its Antioxidant and Antimicrobial Activities
Source: Foods. 2020 Oct 23;9(11):1522. doi: 10.3390/foods9111522 (PMC7690723; doi:10.3390/foods9111522)
Supplement: Supplementary file 1 [file foods-09-01522-s001.pdf]

Supplementary Material

Compositional Features of 'Kweli' Red Raspberry and its Antioxidant and Antimicrobial Activities

Ana Luísa Vara, José Pinela, Maria Inês Dias, Jovana Petrović, António Nogueira, Marina Soković, Isabel C.F.R. Ferreira and Lillian Barros

Table S1. Fatty acids composition of 'Kweli' red raspberry.

Fatty acids and relative ratios	Relative percentage (%)[1]
Caproic acid (C6:0)	0.72 ± 0.01
Caprylic acid (C8:0)	0.32 ± 0.01
Capric acid (C10:0)	0.58 ± 0.01
Undecanoic acid (C11:0)	1.17 ± 0.02
Lauric acid (C12:0)	1.11 ± 0.02
Myristic acid (C14:0)	1.6 ± 0.02
Pentadecanoic acid (C15:0)	0.45 ± 0.01
Palmitic acid (C16:0)	20.2 ± 0.3
Palmitoleic acid (C16:1)	1.66 ± 0.01
Heptadecanoic acid (C17:0)	0.452 ± 0.005
Stearic acid (C18:0)	6.25 ± 0.09
Oleic acid (C18:1*n*-9c)	27.1 ± 0.3
Linoleic acid (C18:2*n*-6c)	11.9 ± 0.3
α-Linolenic acid (C18:3*n*-3)	16.6 ± 0.3
Arachidic acid (C20:0)	3.41 ± 0.05
cis-11-Eicosenoic acid (C20:1)	0.49 ± 0.02
Eicosenoic acid (C21:0)	0.35 ± 0.01
Behenic acid (C22:0)	3.48 ± 0.07
cis-13,16-Docosadienoic acid (C22:2)	0.66 ± 0.02
Tricosanoic acid (C23:0)	0.46 ± 0.01
Lignoceric acid (C24:0)	1.17 ± 0.02
Saturated fatty acids (SFA)	41.7 ± 0.1
Monounsaturated fatty acids (MUFA)	29.2 ± 0.3
Polyunsaturated fatty acids (PUFA)	29.1 ± 0.7
PUFA/SFA ratio	0.70 ± 0.02
PUFA *n*-3/*n*-6 ratio	1.40 ± 0.01

[1]The results are presented as mean ± standard deviation.
